# Excess Mortality Among US Physicians During the COVID-19 Pandemic

**DOI:** 10.1001/jamainternmed.2022.6308

**Published:** 2023-02-06

**Authors:** Mathew V. Kiang, Lindsey E. Carlasare, Sonoo Thadaney Israni, John J. Norcini, Junaid A. B. Zaman, Kirsten Bibbins-Domingo

**Affiliations:** 1 Epidemiology and Population Health, Stanford University School of Medicine, Stanford, California; 2 American Medical Association, Chicago, Illinois; 3 PRESENCE Center, Stanford University School of Medicine, Stanford, California; 4 Department of Cardiovascular Medicine, University of Southern California, Los Angeles; 5 Editor in Chief, *JAMA* and the JAMA Network, Chicago, Illinois

## Abstract

This cross-sectional study examines the death rates among active and nonactive physicians aged 45 to 84 years.

In the US, the COVID-19 pandemic has already resulted in over 1 million excess deaths,^1^ defined as the difference between the number of observed and expected deaths over a specified period.^2^ Despite their essential role in the pandemic response, little is known about excess deaths among physicians. Using data from the American Medical Association (AMA), we calculated excess deaths from March 2020 through December 2021 among US physicians.

## Methods

Using the AMA Masterfile and corresponding Deceased Physician File, we fit quasi-Poisson models, accounting for within-year seasonality and long-term trends,^3^ to estimate monthly mortality from January 2016 through February 2020 among physicians aged 45 to 84 years. We used this counterfactual model to estimate expected deaths from March 2020 through December 2021, and we calculated differences between observed and expected deaths to identify excess deaths. Excess mortality estimates were annualized to excess deaths per 100 000 person-years. We analyzed the total sample by age group and type of practice. We fit alternative model specifications in sensitivity analyses. For comparison, we calculated excess deaths in the US general population (eMethods in Supplement 1). We excluded younger physicians because this group experienced fewer than 5 deaths per month during the period of interest. All analyses were performed using R 4.2.1 (R Foundation for Statistical Computing). The Stanford University Institutional Review Board approved the study. The Stanford Administrative Panels on Human Subjects Research waived the informed consent requirement for various reasons. We followed the STROBE reporting guideline.

## Results

From March 2020 through December 2021, there were 4511 deaths (representing 622 [95% CI, 476-769] more deaths than expected) among a monthly mean (SD) of 785 631 (8293.5) physicians. These physicians consisted of 34.7% females and 65.3% males aged 45 to 84 years (Table). There were 43 (95% CI, 33-53) excess deaths per 100 000 person-years.

**Table.** Number of Mean Monthly Physicians, Observed Deaths, Expected Deaths, and Excess Deaths by Age Group and Type of Practice Among US Physicians From March 2020 Through December 2021

Age group, y	Type of practice	Mean (SD) No. of physicians per mo	Total No. of deaths	No. (95% CI)	
				Expected deaths	Excess deaths
45-64	Active physician providing direct patient care	426 015 (262.0)	652	571 (516 to 625)	81 (27 to 136)
45-64	Active physician not providing direct patient care	37 648 (781.7)	39	26 (13 to 38)	13 (1 to 26)
45-64	Nonactive physician	12 121 (743.5)	47	36 (22 to 50)	11 (–3 to 25)
65-74	Active physician providing direct patient care	133 743 (4059.8)	750	642 (587 to 698)	108 (52 to 163)
65-74	Active physician not providing direct patient care	16 229 (254.6)	73	81 (60 to 102)	–8 (–29 to 13)
65-74	Nonactive physician	54 612 (173.2)	511	420 (375 to 465)	91 (46 to 136)
75-84	Active physician providing direct patient care	25 524 (1351.2)	403	318 (278 to 357)	85 (46 to 125)
75-84	Active physician not providing direct patient care	6268 (269.6)	113	103 (81 to 125)	10 (–12 to 32)
75-84	Nonactive physician	73 470 (1976.0)	1923	1695 (1601 to 1789)	228 (134 to 322)
All: 45-84	Active physician providing direct patient care	585 283 (5658.6)	1805	1520 (1427 to 1613)	285 (192 to 378)
All: 45-84	Active physician not providing direct patient care	60 144 (1293.2)	225	201 (169 to 233)	24 (–8 to 56)
All: 45-84	Nonactive physician	140 204 (1423.0)	2481	2120 (2015 to 2226)	361 (255 to 466)

There was a strong age gradient among active physicians providing direct patient care, with excess deaths per 100 000 person-years of 10 (95% CI, 3-17) in the youngest group and 182 (95% CI, 98-267) in the oldest group (Figure, A). Within all age groups, physicians had substantially lower excess mortality than the general population (Figure, A). Nonactive physicians had the highest excess deaths per 100 000 person-years (140; 95% CI, 100-181) compared with active physicians providing direct patient care (27; 95% CI, 18-35) and active physicians not providing direct patient care (22; 95% CI, –8 to 51) but a substantially lower excess mortality rate than the general population (294; 95% CI, 292-296).

**Figure.**
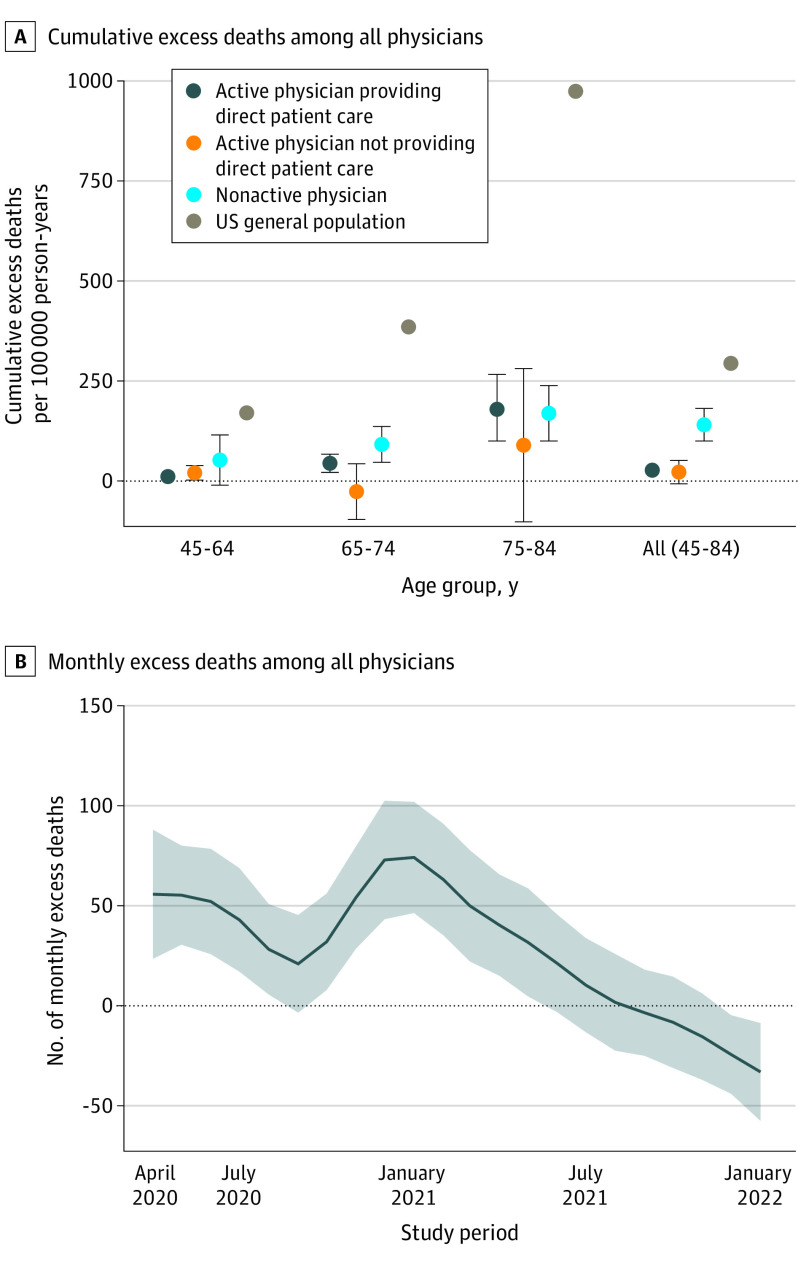
Excess Mortality Among Physicians From March 2020 Through December 2021 A, Error bars represent 95% CIs of the excess death estimates. For reference, excess deaths in the US general population over the same period were included. B, The shaded area represents the 95% CI of the excess death estimate.

Among all active physicians, excess deaths peaked to over 70 in December 2020 and then had a rapid monotonic decrease in 2021. There was no statistically significant excess mortality after April 2021 (Figure, B). These results were robust to alternative model specifications.

## Discussion

From March 2020 through December 2021, US physicians experienced 622 more deaths than expected. There were no excess deaths among physicians after April 2021, coinciding with the widespread availability of COVID-19 vaccines. Across age groups, physicians had substantially lower excess mortality than the general population; however, active physicians had lower excess mortality than nonactive physicians despite their higher risk of contracting SARS-CoV-2 infection.^4^ The findings suggest that personal protective equipment use, vaccine requirements, infection prevention protocols, adequate staffing, and other workplace-based protective measures were effective in preventing excess mortality. Additionally, increased excess deaths among older active physicians providing direct patient care suggest that workplace policies should prioritize mitigating risk in this group.

Study limitations include unidentified physician deaths and pandemic-related workforce changes, such as early retirement among older physicians, which would result in underestimation of mortality. During the first year of the pandemic, US physicians experienced excess mortality in addition to increased workplace stress and burnout.^5^ During COVID-19 surges, these conditions may strain hospitals, resulting in excess deaths in the general population.^6^ Preventing excess deaths among physicians is an important component of mitigating excess deaths in the general population.

## Notes

Supplement.
eMethods

eReferences

Supplement 2.
Data Sharing Statement
