# Supplementary material for: Excess Mortality Among US Physicians During the COVID-19 Pandemic
Source: JAMA Intern Med. 2023 Feb 6;183(4):374–6. doi: 10.1001/jamainternmed.2022.6308 (PMC10071333; doi:10.1001/jamainternmed.2022.6308)
Supplement: Supplement. — eMethods eReferences [file jamainternmed-e226308-s001.pdf]

Supplementary Online Content

Kiang MV, Carlasare LE, Thadaney Israni S, Norcini JJ, Zaman JAB, Bibbins-Domingo K. Excess mortality among US physicians during the COVID-19 pandemic. *JAMA Intern Med.* Published online February 6, 2023. doi:10.1001/jamainternmed.2022.6308

eMethods

eReferences

This supplementary material has been provided by the authors to give readers additional information about their work.

© 2023 Kiang M. et al. *JAMA Internal Medicine.*

eMethods

Here, we describe the data files, definitions, and statistical analyses in more detail.

AMA Physician Masterfile. For our denominator, we use data from the American Medical Association (AMA). The AMA has collected and maintained data on physicians since 1906. The AMA Physician Masterfile includes current and historical education, training, demographic (such as age, sex, and race/ethnicity), and practice data for more than 1.4 million physicians, residents, and medical students in the United States. Specifically, a record is established when individuals enter a medical school accredited by the Liaison Committee on Medical Education (LCME) or in the case of international medical graduates, upon entry into a post graduate training program accredited by the Accreditation Council for Graduate Medical Education (ACGME). A record for osteopathic medical students and physicians is created upon confirmation of attendance or completion of an accredited osteopathic medical school, entry into a post graduate training program accredited by ACGME, or upon licensure by a US medical licensing board. Data on residents are gathered and verified through annual surveys to all ACGME programs. Additional information is collected from authoritative data sources including licensing boards, the American Board of Medical Specialties (ABMS), and the National Plan and Provider Enumeration System (NPPES). The AMA also conducts outbound phone calls and surveys to over 350,000 physicians annually to verify information. In addition, physicians and practices can contact the AMA to update or correct information. Data in the AMA Physician Masterfile are updated monthly and harmonized to create an end-of-year file.

In addition, the AMA categorizes the primary practice of providers in a field labeled “type of practice” based on data gathered for the Masterfile (e.g., licensing boards, the DEA, type of office address). We divided providers into three mutually-exclusive categories based on type of practice: (1) actively practicing physicians who provide direct patient care (“direct patient care”), (2) actively practicing physicians who do not provide direct patient care (“medical research”, “administration”, “unclassified”, “non-patient care”, “medical teaching”, and “resident”), or (3) non-active providers (“retired”, “temporarily not in practice”, “semi-retired”, and “not active for other reasons”).

© 2023 Kiang M. et al. *JAMA Internal Medicine.*

From 2015 to 2021, the AMA Physician Masterfile contained a total of 8,673,265 person-years of observation across 1,339,869 unique physicians. By type of practice, there are 7,348,335 person-years among 1,172,786 unique actively practicing physicians and 1,324,930 person-years among 233,136 unique non-actively practicing physicians. After removing physicians who were not between 45 to 84 years of age, there were 4,347,423 person-years of observation among 733,570 unique actively practicing physicians and 948,816 person-years of observation among 187,468 unique non-actively practicing physicians.

AMA Deceased Physician File. Deaths for all physicians in the AMA Physician Masterfile are tracked using several sources including the Social Security Administration's Death Master File and obituary services. These data contain the date of death but do not contain the cause of death. However, during the COVID-19 pandemic, physician deaths were further investigated to determine if SARS-CoV-2 infection was a potential contributor to the death.

From 2016 to 2021, the AMA Deceased Physician File contained a total of 28,531 deaths. Across all deaths, 7,824 were among practicing physicians. Of these, 6,621 deaths were among practicing physicians between 45 and 84 years of age.

Statistical Approach. We used the end-of-year AMA Physician Masterfile to linearly interpolate monthly provider counts. For our statistical analyses, we excluded younger physicians (those under 45 years of age) due to few observed deaths during the period of interest, which would limit the ability to generate reasonable counterfactual models. Specifically, during the period of interest, there were 103 deaths among 25-44 year old physicians (across all types of practice) for an average of 4.7 deaths per month (average monthly crude mortality rate of 1.2 per 100,000) compared to the 45-64 year old age group, which had 738 deaths for an average of 33.6 deaths per month (average monthly crude mortality rate of 8.1 per 100,000). Further, we excluded older physicians due to a small, rapidly shifting denominator such that a linear interpolation between years for each practice type is unlikely to be accurate in this age group. Specifically, during the period of interest, there were an average of 5,151

© 2023 Kiang M. et al. *JAMA Internal Medicine.*

active providers per month in the 85 years and over age group compared to an average of 31,802 active providers per month in the 75 to 84 year old age group. Due to small sample size, we were unable to evaluate the impact by gender, race/ethnicity, or specialty.

Our modeling approach is based on the work of Acosta and Irizarry[1] and has been described in detail in the Supplemental Text S1 of a previous paper by Kiang et al.[2] We briefly describe the approach here. For our counterfactual model, we assume monthly death counts follow an overdispersed quasi-Poisson distribution:

$$Y_t \sim Poisson(\mu_t) \text{ for } t \in I_{< March\ 2020}$$

where Y_t is the observed number of deaths at month t, μ_t is the mean number of deaths at month t, and $I_{< March\ 2020}$ is the set of observations before March 1, 2020. The variance of the distribution is $Var(Y) = \mu\phi$ where ϕ is the dispersion parameter estimated from the data using the `glm(… family = "quasipoisson")` function call in `R` 4.2.1. In this setting, the model is fit using quasi-maximum likelihood estimation.

We then decompose the mean μ_t into two parts:

$$\mu_t = N_t\, exp\{\alpha(t) + s(m_t)\}$$

where $s(m_t)$ is a periodic function of time at month m_t that accounts for within-year seasonality, $\alpha(t)$ is a smooth function of time that accounts for long-term secular trends, and N_t is the population at time t which we treat as an offset. We modeled s as a Fourier basis with 1 to 4 harmonics and α as a natural cubic spline with 0 to 2 internal knots, depending on the subpopulation of interest. We fit stratified models for each age group and type of practice. To identify the optimal set of hyperparameters for our baseline models, we used time series cross validation with an expanding forecasting origin.[3] We fit all models on data from January 1, 2016 through February 29, 2020 and selected the model with the lowest out-of-sample mean squared error. The final model was then used to predict the expected number of deaths after March 1, 2020 for the population of interest. Importantly, data after February 29, 2020 are never used for modeling fitting or evaluation.

© 2023 Kiang M. et al. *JAMA Internal Medicine.*

We used the same approach to estimate excess mortality in the US general population using publicly-available provisional death count data from the CDC National Center for Health Statistics (https://www.cdc.gov/nchs/nvss/vsrr/covid_weekly/index.htm).

All excess death calculations were done in using R 4.2.1 through the `excessmort` package (https://github.com/rafalab/excessmort).[1] After estimating the expected number of deaths, the `excessmort` package then estimates the difference between the observed and expected number of deaths while accounting for natural variability. To estimate annualized excess mortality rates, we took the cumulative excess mortality over the period of interest (March 1, 2020 through December 31, 2021; Figure S2), normalized by the total number of providers at-risk every month, and then annualized to units of 100,000 person-years.

To assess the sensitivity of our results to model selection, we estimated excess mortality using a different, previously-published modeling strategy.[4,5] Specifically, we fit dynamic harmonic regressions (DHR), an extension of autoregressive integrated moving average (ARIMA) models.[6] Similar to our primary analysis, DHR accounts for seasonal trends, long-term trends, and population size; however, unlikely the parametric assumptions of our primary analyses, uncertainty intervals were calculated by bootstrapping 50,000 simulations and taking the 2.5th and 97.5th percentiles as the 95% uncertainty interval. In sensitivity analyses, our results remain qualitatively the same.

Reproducible code is available at: http://github.com/mkiang/excess_physician_mortality. We note that due to our data use agreement, we cannot share data. However, we provide code so researchers with access to the AMA Masterfile and Deceased Physician File can reproduce and extend our analyses.

© 2023 Kiang M. et al. *JAMA Internal Medicine.*

eReferences

1. Acosta RJ, Irizarry RA. A Flexible Statistical Framework for Estimating Excess Mortality. Epidemiology. 2022;33(3):346-353. doi:10.1097/ede.0000000000001445
2. Kiang MV, Acosta RJ, Chen YH, et al. Sociodemographic and geographic disparities in excess fatal drug overdoses during the COVID-19 pandemic in California: A population-based study. The Lancet Regional Health - Americas. 2022;11:100237. doi:10.1016/j.lana.2022.100237
3. Bergmeir C, Hyndman RJ, Koo B. A note on the validity of cross-validation for evaluating autoregressive time series prediction. Comput Stat Data An. 2018;120:70-83. doi:10.1016/j.csda.2017.11.003
4. Chen YH, Glymour MM, Catalano R, et al. Excess Mortality in California During the Coronavirus Disease 2019 Pandemic, March to August 2020. Jama Intern Med. 2021;181(4). doi:10.1001/jamainternmed.2020.7578
5. Chen R, Aschmann HE, Chen YH, et al. Racial and Ethnic Disparities in Estimated Excess Mortality From External Causes in the US, March to December 2020. Jama Intern Med. 2022;182(7). doi:10.1001/jamainternmed.2022.1461
6. Hyndman, R.J., & Athanasopoulos, G. (2018). Section 9.5 Dynamic harmonic regression from Forecasting: principles and practice, 2nd edition, OTexts: Melbourne, Australia. Accessed on July 21, 2022 at https://otexts.com/fpp2/dhr.html.

© 2023 Kiang M. et al. *JAMA Internal Medicine.*
