# Supplementary material for: Excess Mortality Among US Physicians During the COVID-19 Pandemic
Source: JAMA Intern Med. 2023 Feb 6;183(4):374–6. doi: 10.1001/jamainternmed.2022.6308 (PMC10071333; doi:10.1001/jamainternmed.2022.6308)
Supplement: Supplement 2. — Data Sharing Statement [file jamainternmed-e226308-s002.pdf]

Data Sharing Statement

Kiang. Excess Mortality Among US Physicians During the COVID-19 Pandemic. *JAMA Intern Med*. Published February 06, 2023. doi:10.1001/jamainternmed.2022.6308

Data
Data available: No

Additional Information
Explanation for why data not available: In accordance with our data use agreement, physician-level data cannot be publicly shared. We provide reproducible code such that other researchers who have access to the AMA Masterfile and Deceased Physician File can reproduce or extend our analyses. Code is available at: http://github.com/mkiang/excess_physician_mortality.
